# Microsecond simulations to investigate the structural mechanism of super-resistant double mutations in BTK to the covalent inhibitor ibrutinib in multiple leukemia

**DOI:** 10.1038/s41598-025-24745-7

**Published:** 2025-11-20

**Authors:** Abbas Khan, Syed Shujait Ali, Muhammad Ammar Zahid, Fahad M. Alshabrmi, Raed M. Al-Zoubi, Mohanad Shkoor, Anwar Mohammmad, Dong-Qing Wei, Abdelali Agouni

**Affiliations:** 1 Department of Pharmaceutical Sciences, College of Pharmacy, QU Health, Qatar University, P.O. Box 2713, Doha, Qatar; 2 Center for Biotechnology and Microbiology, University of Swat, Swat, Khyber Pakhtunkhwa Pakistan; 3 Department of Medical Laboratories, College of Applied Medical Sciences, Qassim University, 51452 Buraydah, Saudi Arabia; 4 Surgical Research Section, Department of Surgery, Hamad Medical Corporation, Doha, Qatar; 5 Department of Biomedical Sciences, College of Health Sciences, QU Health, Qatar University, P.O. Box 2713, Doha, Qatar; 6 Department of Chemistry, Jordan University of Science and Technology, P.O. Box 3030, Irbid, 22110 Jordan; 7 Department of Chemistry and Earth Sciences, College of Arts and Science, Qatar University, P.O. Box 2713, Doha, Qatar; 8 Precision Health Analysis Unit, Translational Research, Dasman Diabetes institute, Dasman, Kuwait; 9 Department of Biostatistics and Bioinformatics, College of Life Sciences and Biotechnology, Shanghai Jiao Tong University, Shanghai, P.R. China

**Keywords:** Bruton’s tyrosine kinase, BTK, Ibrutinib, Drug resistance, Molecular simulation, Free energy calculation, Biochemistry, Cancer, Computational biology and bioinformatics, Drug discovery, Structural biology

## Abstract

Bruton’s Tyrosine Kinase (BTK) is an anchor in B-cell receptor signaling and plays an important role in chronic lymphocytic leukemia (CLL). The use of covalent inhibitors of BTK, such as ibrutinib, enhances the survival of patients with CLL. However, mutations at the C481 residue cause resistance to ibrutinib and diminish its clinical efficacy. Recently, super-resistant mutants, i.e., T474M-C481S and T474I-C481S, were reported to cause manifold resistance to the BTK-targeting drug, ibrutinib; however, the mechanism of this resistance is still elusive. Structure-based approaches proved to be effective in deciphering drug resistance mechanisms (s) that could guide the development of novel, effective therapeutics. Therefore, we used molecular modeling combined with biophysical simulation approaches to determine the impact of T474M-C481S and T474I-C481S mutations on the binding of ibrutinib. Our results revealed that essential hydrogen bonds and a covalent interaction with C481 are lost due to these mutations. Using µs simulations, our results revealed that the regions 432–439 and 545–559 demonstrated dynamically unstable behavior with the transition of secondary structure, where a helix to loop and loop to helix transition could be observed. Structural compactness, residue flexibility, and average hydrogen bonds in each trajectory reported significant variations. The binding free energy calculation using MM-GBSA (Molecular Mechanics Generalized Born Surface Area) and MM-PBSA (Molecular Mechanics Poisson–Boltzmann Surface Area) approaches revealed that both the vdW and electrostatic energies are reduced in mutants. Using the MM-PBSA approach, the wild type demonstrated a total binding free energy (TBE) of -42.65 ± 0.08 kcal/mol, while T474M-C481S and T474I-C481S had TBE values of -38.81 ± 0.18 kcal/mol, and − 33.04 ± 0.13 kcal/mol, respectively. The MM-GBSA results revealed that the wild type had a TBE of -60.33 ± 0.06 kcal/mol, while the TBE values for T474M-C481S and T474M-C481S mutants were − 53.18 ± 0.12 kcal/mol and − 49.12 ± 0.10 kcal/mol, respectively. PCA and FEL results further revealed the dynamic variations caused by these mutations. These findings underline the significant impact of mutations T474M and C481S on the binding free energy, highlighting the importance of these residues in ibrutinib-BTK interactions.

**Supplementary Information:**

The online version contains supplementary material available at 10.1038/s41598-025-24745-7.

## Introduction

The member of the Tec kinase family, Bruton’s Tyrosine Kinase (BTK), plays a vital and crucial role in B-cell receptor (BCR) signalling^1,2^. Following the stimulation of BCR, the activation of BTK at the cell membrane facilitates the phosphorylation of downstream targets and initiation of essential signalling cascades for the development and function of B-cells such as B lymphocyte proliferation, regulation, differentiation, and antibody production^3,4^. BTK is an anchor in BCR signalling and plays an important role in cellular responses that are associated with immune function. Its association and linkage in numerous B-cell abnormalities and malignancies, such as chronic lymphocytic leukemia (CLL), small lymphocytic leukemia (SLL), mantle cell lymphoma, diffuse large cell lymphoma (DLBCL), and Waldenstrom’s macroglobulinemia (WM), make it a valuable target for the development of novel therapeutic agents for various malignancies^5,6^. Similarly, aberrations in signaling cascades linked with BTK enhance uncontrolled B-cell proliferation, and further investigation along these lines on BTK will thus be useful for the development of novel targeted cancer therapies.

Bruton’s tyrosine kinase (BTK) has been recognized as a validated target in B-cell malignancies, and several small-molecule inhibitors have been developed to inhibit BTK kinase activity. Among these inhibitors, the covalent BTK inhibitors that have progressed furthest in the clinic include ibrutinib, acalabrutinib, and zanubrutinib; all three of which bind irreversibly to the C481 residue in the ATP-binding pocket and inhibit kinase activity^7–9^. These agents have shown impressive clinical activity in chronic lymphocytic leukemia (CLL) and mantle cell lymphoma (MCL), making BTK inhibition the preferred front-line therapy. However, the need for covalent modification of C481 makes these drugs susceptible to mutations that confer drug resistance, particularly the C481S mutation that abrogates irreversible modification and mutations in gatekeeper or compound residues (e.g., T474 + C481) that alter the binding pocket and lower the efficacy of the drug^10,11^. Therefore, to inform future development of next-generation BTK inhibitors, it is critical to characterize the structural basis of both covalent compound binding and mutations that confer resistance. The advent of small molecules that act as covalent inhibitors, such as ibrutinib, ushered in a new era for the effective management of these malignancies. New small molecules or second-generation BTK inhibitors such as acalabrutinib, tirabrutinib, zanubrutinib, and orelabrutinib were also launched for the treatment of BTK-associated abnormalities^12^. The introduction of these small BTK inhibitors changed the paradigm of cancer therapy for various leukemias. These inhibitors bind to the cysteine 481 (C481) residue of the kinase domain, leading to the inhibition of BTK activation and thus interfering with BCR signalling^13,14^. These activities are helpful to effectively control cell proliferation and also initiate apoptosis in malignant B cells. These inhibitors were promising and were found effective at suppressing cell proliferation; however, the emergence of resistance due to numerous mutations posed serious threats to the clinical efficacy of these inhibitors. Excessive use of these inhibitors confers resistance against them and can lead to disease progression.

As mentioned above, the remarkable efficacy of these inhibitors and the threat posed by the development of resistance undermine the long-term use of these targeted therapies. To understand the mechanism(s) of resistance development, several studies were conducted^15^. These molecular-based investigations suggest a role for changes in the BTK gatekeeper residue, along with substitutions in the C481 residue. This gatekeeper residue (a conserved threonine, T474) in the BTK domain is linked with the maintenance of the structural integrity of the kinase pocket and the facilitation of the binding of irreversible inhibitors. The changes and alterations in the conformation of this vital residue due to mutations, especially point mutations, reduce the efficacy of therapeutic agents targeting BTK^16^.

The use of covalent inhibitors such as ibrutinib to target BTK enhances the survival chances of CLL patients. This covalent interaction between ibrutinib and the C481 residue of BTK provided desirable pharmacological outcomes^17^. However, the efficacy of ibrutinib was diminished by the mutation at the C481 residue, leading to the development of resistance and a poor clinical response. Attempts to counter the conferment of resistance to covalent inhibitors (e.g., ibrutinib) led to the development of non-covalent inhibitors^18^. Non-covalent inhibitors do not bind the C481 residue; therefore, they became an alternative to covalent inhibitors for the control of malignancies associated with B cells^19,20^. However, mutations occurring at other sites than C481 were found to be crucial in limiting the efficacy of non-covalent inhibitors by conferring resistance against them. For instance, the non-C481 mutations such as V416L, A428D, T474I, M437R, and L528W were reported to confer resistance to both covalent and non-covalent inhibitors. Similarly, a recent study reported the role of these mutations in conferring resistance to covalent and non-covalent inhibitors such as ibrutinib, acalabrutinib, zanubrutinib, pirtobrutinib, ARQ-531, vecabrutinib, and fenebrutinib. Based on the *K*_*inact*_*/Ki* values, mutations A428D and L528W completely stop the binding of ibrutinib to BTK, whereas the effect of other mutations (T474I, M437R, and V416L) is not pronounced on covalent drugs^21–23^. Moreover, double mutations, i.e., T474I-C481S and T474M-C481S, termed as super-resistant, are reported to cause manifold resistance to ibrutinib and pose new challenges to the existing chemotherapy against B-cell leukemia^24,25^. Therefore, it is highly recommended to conduct extensive research to decipher the role of these mutations in the development of drug resistance and search for novel inhibitors to evade this resistance for the treatment of various malignancies.

Relying on structure-based computational approaches will greatly contribute to the understanding of resistance mechanisms as well, and it would be helpful to ascertain the effect of mutations on drug binding. A previous study by Khan et al^26^. followed this strategy to determine the impact of mutations conferring resistance to enzalutamide in prostate cancer treatment^26^. The mutations in the estrogen receptor conferring resistance to the active ligands were thoroughly studied, and it was reported that the flexibility of certain loops that cause the release of the drugs could explain the mechanism of resistance. In the current study, computational approaches will be explored to investigate the impact of T474M-C481S and T474I-C481S double mutations on the binding of ibrutinib. This study was conducted to check the role of these mutations on the binding of the drug to BTK. Molecular docking and computational molecular simulation were used for mutant complexes. We have explicitly emphasized the extended microsecond-scale MD analysis of clinically relevant double BTK mutations (T474M–C481S and T474I–C481S) against ibrutinib. Moreover, by integrating binding free energy calculations with essential dynamics and conformational landscape analysis, we uncover the structural and energetic mechanisms underlying the synergistic resistance phenotype. This combination of longer timescale simulations, multiple resistance mutations, and advanced post-simulation analyses establishes a distinct and novel contribution compared to existing literature. The current study explores the mechanism of drug resistance to ibrutinib, which can be used for the development of novel and more effective inhibitors against BTK for improved clinical outcomes.

## Materials and methods

### Structure retrieval and preparation

The protein and ibrutinib complex coordinates were retrieved from the protein structural data repository (RCSB) with the help of accession number 5P9J^27^. These structures were prepared and minimized for the simulation. Dunbrack 2010 backbone-dependent rotamer library was used for the creation of mutants such as T474M-C481S and T474I-C481S. The DOCKPREP option was applied for the complex preparation, and incomplete side chains were replaced with the Dunbrack 2010 library, whereas the addition of hydrogens and correction of charges were performed in the Chimera software^28^. Loop modeler with the Chimera was used for the modeling of missing residues. Then, two steps of minimization, i.e., steepest descent for 100 steps with a step size of 0.02 Å and conjugate gradient minimization of 10 steps with the same step size, were performed. These prepared structures were used for molecular dynamics simulation to evaluate the effect of T474M-C481S and T474I-C481S on the structure and binding of ibrutinib.

### Molecular dynamics simulation (MDS) of the wild-type and mutants

All the complexes obtained after molecular docking were subjected to molecular dynamics simulation (MDS) for the understanding of the dynamic behavior of these complexes. The best complexes [wild type (WT), T474M-C481S, and T474I-C481S] were subjected to additional analysis. Dynamic stability and various other parameters were estimated as a result of this analysis with the help of the FF19SB force field, utilizing the AMBER20 simulation package and AMBERtools21. The ligand topology was constructed with an antechamber using GAFF2 (General Amber Force Field 2)^29–33^. The balance between speed and precision was obtained by adding a rectangular box “optimal point charge” (OPC), the size of each side was 14.0 Å, for solvating each complex. The neutralization was performed by the addition of sodium ions to each complex. The relaxation of the system to avoid undesirable atomic collisions was obtained by employing the steepest descent and conjugate gradient techniques to minimize the complexes. Each system was heated up to 300 K and then allowed to equilibrate for 2 ns with weak restraints. Afterward, a 1000 ns production run was performed. Langevin thermostats and Particle Mesh Ewald algorithms were employed for temperature regulation and addressing of long-range electrostatic interactions, respectively. At the same time, the management of covalent bonds was performed on the SHAKE algorithm. GPU-accelerated PMEMD.CUDA approach was adopted to accelerate the simulation of each complex^34^. Within our framework, for the wild-type BTK model, we explicitly modeled the irreversible covalent bond formation between C481 and ibrutinib in our simulations by parameterizing the covalent adduct. This was accomplished by creating the ligand-residue complex in Schrödinger Maestro and then parameterizing the modified cysteine-ibrutinib adduct using Antechamber/GAFF with AM1-BCC charges, such that we treated the C481-ligand bond as a rigidly fixed covalent bond throughout each MD simulation. Additionally, the mutant systems (C481S double mutants) were modeled without covalent bond formation, as the reactive cysteine was absent, producing models where the inhibitors were only considered non-covalent binders present in the active site. CPPTRAJ and PTRAJ modules facilitate the processing of the trajectories^35^. Further details are provided in the supplementary materials.

### Interaction analysis of the relaxed complexes

The impact of each mutation on the binding of ibrutinib, the stable portion of the simulation was used to obtain a single structure and was subjected to 2D and 3D interactions analysis. Each complex binding mode was observed in PyMOL and the academic version of Schrodinger Maestro was used to analyze the binding of each complex^36,37^. The binding variations were compared with the post-simulation structures from each complex.

### Binding-free energy estimation

The advantages of the binding free energy calculation were utilized to check the binding strength between the interacting partners. This approach is useful to computationally determine the real-time binding of the interacting molecules accurately. With the help of Molecular Mechanics Poisson−Boltzmann Surface Area (MM/PBSA) and Molecular Mechanics Generalized Born surface area (MM/GBSA) methods, these complexes were re-evaluated^38^. These methods successfully assess the impact of specific mutations on drug resistance and estimate binding energies^26,38–40^. The same methods were used for the calculation of the overall binding free energy of the WT and mutant-BTK complexes. The following equation was used to calculate each term in the total binding energy.

1 $$\:{\varDelta\:G}_{bind}=\:{G}_{(complex,\:\:\:solvated)}-\:{G}_{\left(BTK,\:\:\:solvated\right)}-\:{G}_{(pirtobrutinib,\:\:\:solvated)}$$

Further details are provided in the supplementary materials.

### Essential dynamics

PCA is a method frequently used to analyze data from simulations of biological macromolecules obtained by molecular dynamics. It makes qualitative and quantitative analysis simpler and more complete by lowering the dimension of their configurational space. The widespread popularity of PCA is a result of its simplicity of use and somewhat low processing needs. Global translation and rotation’s effects on the coordinates collection are eliminated via PCA^41,42^. The covariance matrix is computed in the second phase using the aligned and centered coordinates. The primary components of the system are then identified by determining the eigenvectors and eigenvalues of the covariance matrix. These eigenvectors correspond to the highest eigenvalues. The system’s most significant movements and structural changes are represented by these key elements, and may be used to examine the trajectory and visualize it. Further details are provided in the supplementary materials.

## Results and discussion

### Structure retrieval and binding analysis of the BTK-ibrutinib complex

**Fig. 1:**
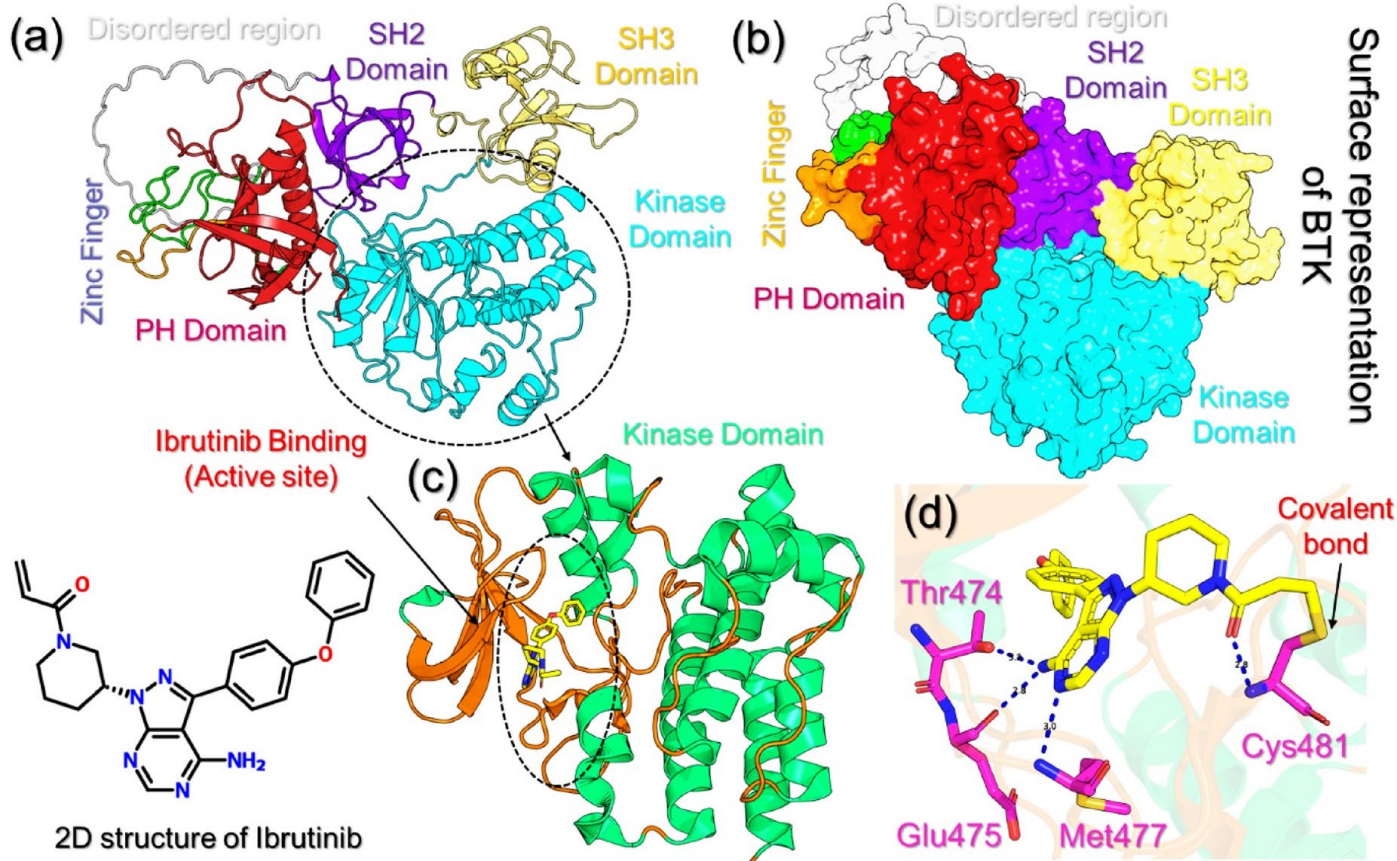
Structure and binding analysis of BTK and ibrutinib. **(a**,** b)** cartoon (a) and surface representation (b) of the structure of BTK and its domains. **(c)** shows the binding of ibrutinib with the kinase domain, while **(d)** shows the 3D interaction pattern of ibrutinib with BTK. The 2D structure of ibrutinib is also shown.

Bruton’s tyrosine Kinase (BTK) is a multidomain protein composed of the pleckstrin homology (PH), Tec homology (TH), SH3 domain, SH2 domain, and kinase domains. The kinase domain is the location of the catalytic site for ATP and covalent inhibitors like ibrutinib. The conserved cysteine C481 residue within this domain serves as the covalent anchor site for irreversible inhibitors, whereas the gatekeeper residue T474 limits accessibility to the hydrophobic pocket, which is positioned just adjacent to the ATP-binding cleft. Other regulatory regions, such as the activation loop (residues 545–559) and the β3–αC loop (residues 432–439), have important functions during active and inactive conformational stabilization. Additionally, mutations within these regions can change the architecture of the binding site and have been demonstrated to confer clinical resistance. The cartoon representation of BTK is depicted in Fig. 1a, while the surface representation of BTK and its different domains is provided in Fig. 1b. The 2-dimensional (2D) structure of ibrutinib is also shown in Fig. 1. The kinase domain interacting with ibrutinib is represented in Fig. 1c, while the 3D interaction pattern of ibrutinib and the BTK kinase domain is depicted in Fig. 1d. Among the hydrogen bonding residues, Thr474, Glu475, Met477, and Cys481 are shown to be involved. Interestingly, it can be noted that Cys481 covalently interacts with ibrutinib, which drives its pharmacological strength in leukemia.

### Post-simulation interaction analysis

**Fig. 2:**
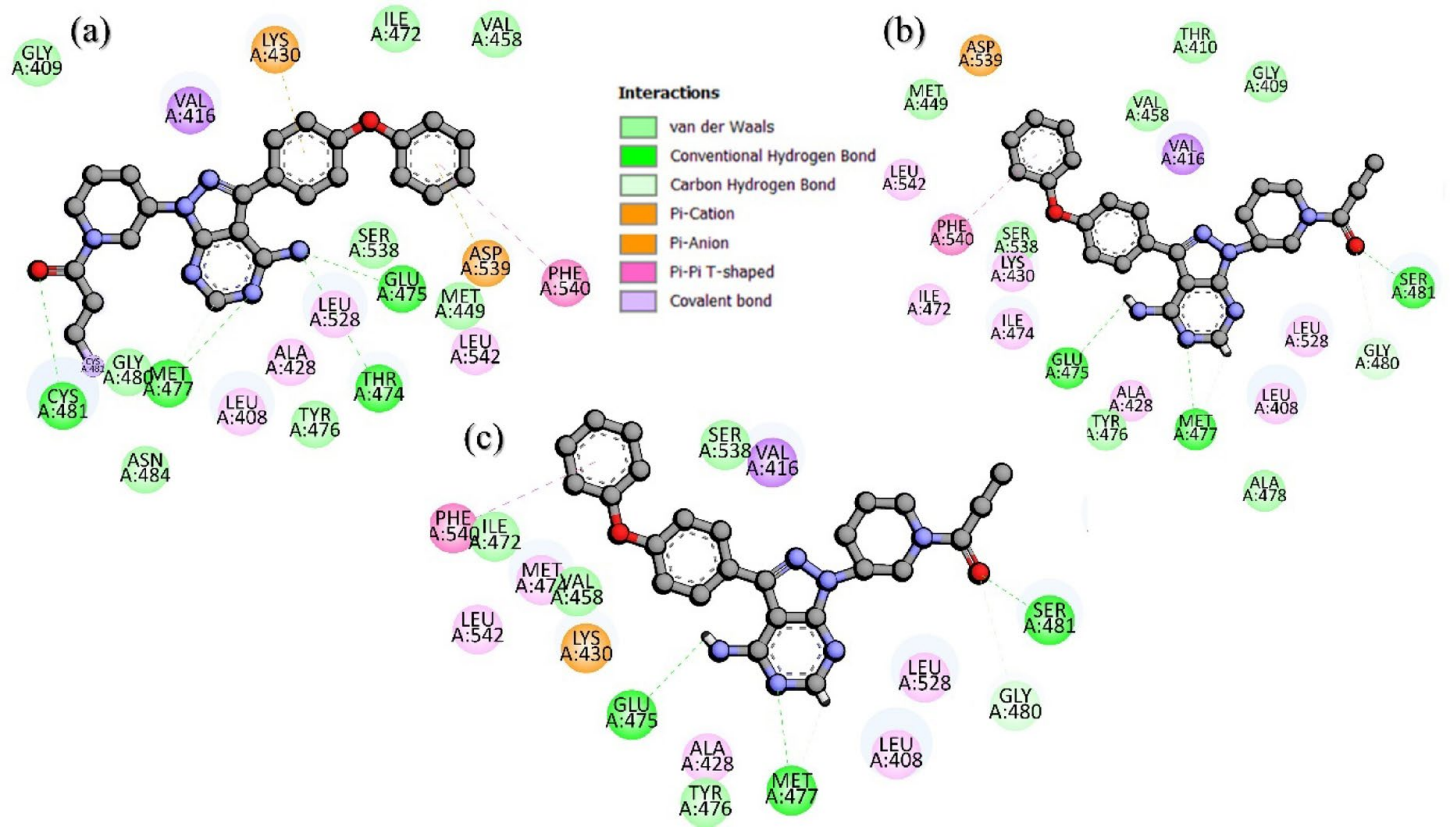
Binding mode of ibrutinib with the WT and mutant BTK. **(a)** shows the binding of ibrutinib with the WT. **(b**,** c)** show the binding of BTK mutants with ibrutinib.

A thorough investigation of the WT and mutant complexes’ interactions was performed on the average structure obtained from MD simulations to identify the binding variations caused by the induced mutations. To determine the binding differences due to mutation, an in-depth analysis of the interactions of the WT and mutant complexes was conducted on the average structure from the MD simulations. For each complex, we searched for the standard hydrogen bonds, hydrophobic interactions, covalent bonds, and other interactions while determining the binding pattern of each complex. As indicated in Fig. 1, a comparative activity profile of ibrutinib against the WT, T474M, and C481S BTK shows a clear reduction in activity when these mutations are introduced. The WT protein exhibited the most inhibition, while the T474M mutant saw a dramatic drop-off in activity, and the C481S mutant further impaired activity. These results provide a confident functional relationship among the structural changes observed in MD simulations and the known experimental drug resistance phenotypes. Similarly, Fig. 2a shows that the WT had four hydrogen bonds, one covalent bond, one π-π, one π-cation/π-anion, and one π-π interaction with Ibrutinib. In contrast, the T474I-C481S mutant complex **(**Fig. 2b**)** had only three hydrogen bonds and one π-π/π-cation interaction. Fewer and weaker interactions indicate weaker drug binding. The T474M-C481S mutant complex **(**Fig. 2c**)** had a similarly smaller number of supporting interactions. In conclusion, the loss of hydrogen bonds, hydrophobic contacts, and π-based interactions is consistent with the loss of efficacy of ibrutinib against the mutant BTK forms. These data demonstrate that the loss of binding interactions due to mutations is the underlying cause of the loss of efficacy of ibrutinib in resistant BTK variants.

### Structural-dynamic features of the WT and mutant complexes

**Fig. 3:**
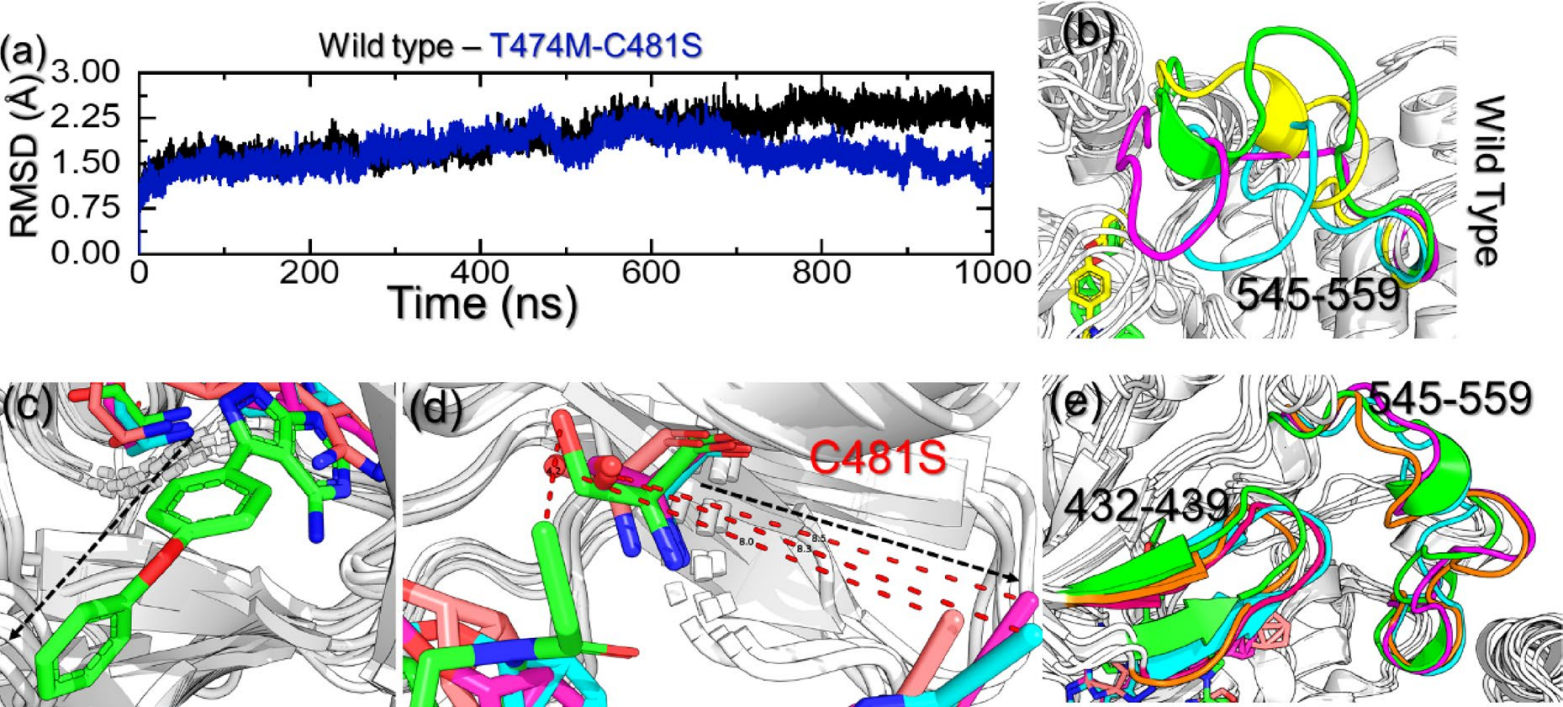
Structural stability graph and essential regions that contribute to the dynamics of the WT and T474M-C481S mutant in complex with ibrutinib. **(a)** shows the RMSD graphs for the WT and the T474M-C481S mutant. **(b)** shows the highly unstable regions in the WT system, **(c)** shows the native structure and simulation structures that determine the differences in the ligand within the cavity, **(d)** shows the distance between the tail of ibrutinib and C481S residue while **(e)** shows the dynamically unstable regions i.e., 432–439 and 545–559 in the T474M-C481S mutant complex during the simulation.

In order to decipher the impact of the binding of ibrutinib and the mutant BTK, we calculated the structural stability as a function of time using the simulation trajectory. The root mean square deviation (RMSD) was computed for each system to understand the dynamic stability patterns during the µs simulation time period. The RMSD for the WT and the T474M-C481S mutant is given in Fig. 3a. As shown in Fig. 3a, both systems were found to converge with each other soon after the system equilibrated, thus demonstrating the validity of our results and showing that a similar atomic path was followed by both systems. The RMSD for both complexes maintained a level of 1.50 Å with a gradual increase during the first 450ns. Afterward, the RMSD differences between WT and mutant became more obvious. It can be noted that the RMSD of the WT remains stable with a gradual increase, with no significant structural perturbations observed. However, the RMSD for the T474M-C481S mutant showed an abrupt decline at 450ns, followed by another steeper decline phase at 600ns that continued until the end of the simulation. Interestingly, the mutant complex exhibited a lower RMSD level than the WT, which demonstrates that the binding of ibrutinib induces structural perturbations and therefore the WT had a higher RMSD than the mutant. We retrieved the highly perturbed frames from the simulation trajectory of the calculated RMSD difference by comparing them with the native structure. The T474M-C481S mutant showed major variations at 468ns, 899ns, 907ns, and 987ns. The structure at 468ns reported an RMSD difference of 1.042 Å in contrast to the native structure, while lower RMSD variations were then observed. The time period 899ns reported a structural deviation of 1.028 Å, while at time points of 907ns and 987ns, the RMSD difference was calculated to be 1.032 Å. This underscores the impact of the induced mutation on the structural stability of the BTK. To identify which regions primarily contribute to the variations in the RMSD values, the respective frames at the aforementioned periods were retrieved and compared with the native structure. For the WT, the highly dynamic time points were 230ns, 685ns, and 968ns, where major deviations were observed. In the case of the WT, the regions 545–559 were observed to move freely and therefore affect the stability of the system. It can be noted that the ligand is stably bound inside the cavity within an acceptable range of contacts **(**Fig. 3b**)**. The two tails are jointly held by key interactions and therefore produce a desirable pharmacological potential. It can be observed that the outer tail is strongly held by a strong covalent bond, which makes it accessible for the residues to establish active contacts and therefore remain stable during the simulation. In the case of the T474M-C481S system, although structurally similar, the ligand was observed to be moved out of the cavity. Figure 3c shows that the initial structure shown in green stably reported the ligand inside the cavity; however, with time, the length between C481S and the ligand increased, consequently increasing the distance between the ligand and this particular residue, making it inaccessible for establishing direct contacts with the protein. This loss of covalent bonds had another consequence of freeing the ligand to move out of the cavity and thus making the other tail inaccessible for the other interactions. The distance between C481S and ibrutinib is indicated in Fig. 3d. Furthermore, unlike the WT, in the mutant, multiple regions were observed to induce structural destabilization. For instance, the regions 432–439 and 545–559 demonstrated a dynamically unstable behavior with the transition of secondary structure, where a helix to loop and loop to helix transition could be observed. The structurally mapped highly dynamic regions are provided in Fig. 3e. Overall, this shows that these two mutations make the ligand inaccessible for the other residues upon the loss of essential covalent contacts and increase the volume of the cavity, allowing free movement and therefore altered conformational ensembles consistent with resistance.

**Fig. 4:**
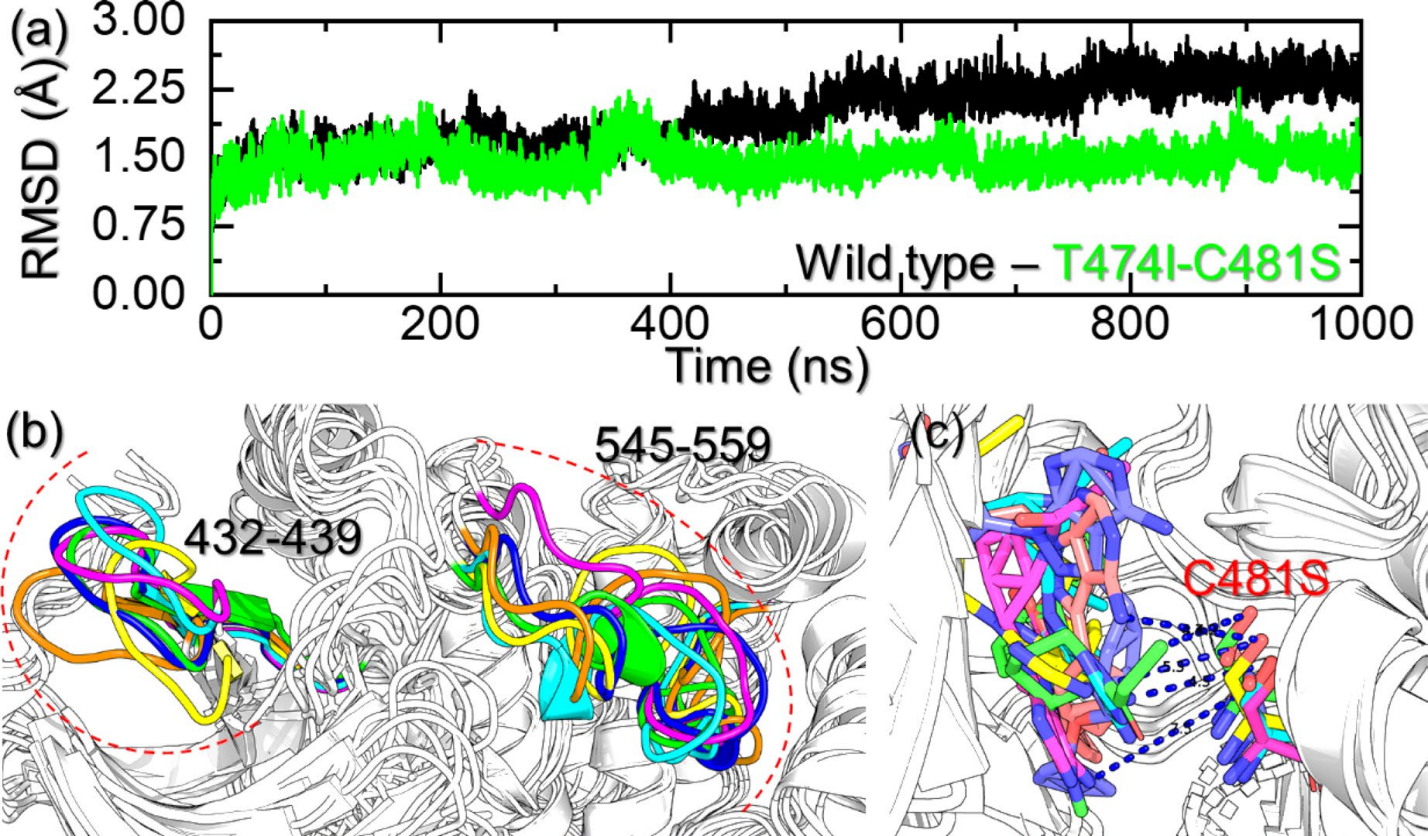
Structural stability graph and essential regions that contribute to the dynamics of the WT and T474I-C481S mutant in complex with ibrutinib. **(a)** shows the RMSD graphs for the WT and the T474I-C481S mutant. **(b)** shows the dynamically unstable regions, i.e., 432–439 and 545–559, in the T474M-C481S mutant complex during the simulation, while **(c)** shows the distance between the tail of ibrutinib and the C481S residue.

On the other hand, the T474I-C481S mutant reported dynamically similar behavior to the T474M-C481S mutant but distinct from the WT. The complex converged with the WT showing a similar atomic configuration until 350ns. The complex then demonstrated a higher RMSD between 360-400ns and then declined. This pattern was consistent until 690ns, then a further decline was observed. The average RMSD was observed to be higher than the WT. At 182ns, 363ns, 631ns, 894ns, and 983ns time points, major deviations in the RMSD patterns were observed. The RMSD difference at 182ns was calculated to be 1.45 Å when compared with the native structure. Moreover, a 1.66 Å RMSD difference was calculated between the native structure and a snapshot at 363ns. Afterward, the RMSD difference was reduced and was calculated to be 1.17 Å, 1.18 Å, and 1.18 Å at 631ns, 894ns, and 983ns timepoints, respectively. This shows the variations in the altered conformational ensembles consistent with resistance during the simulation due to the acquired mutation in the BTK. The RMSD graphs for the WT and the T474I-C481S mutant are given in Fig. 4a. We then analyzed the structural snapshots at this particular time point, and a similar pattern was observed in the trajectory. As shown in Fig. 4b, in the T474M-C481S, the regions 432–439 and 545–559 are dynamically more unstable, which were also reported here in this complex. Furthermore, a distance between the tail of the ligand and the C481S residue was also observed. Although this distance was shorter than the one observed for the T474M-C481S mutant, a similar pattern of dynamic behavior was observed. Therefore, both mutant systems demonstrate similar behavior to induce resistance to ibrutinib. Thus, RMSD combined with structural analyses highlights that the double mutants adopt altered conformational ensembles consistent with resistance. The results obtained from the structural analysis of the trajectories’ snapshots are depicted in Fig. 4b and c.

### Structural compactness analysis

**Fig. 5:**
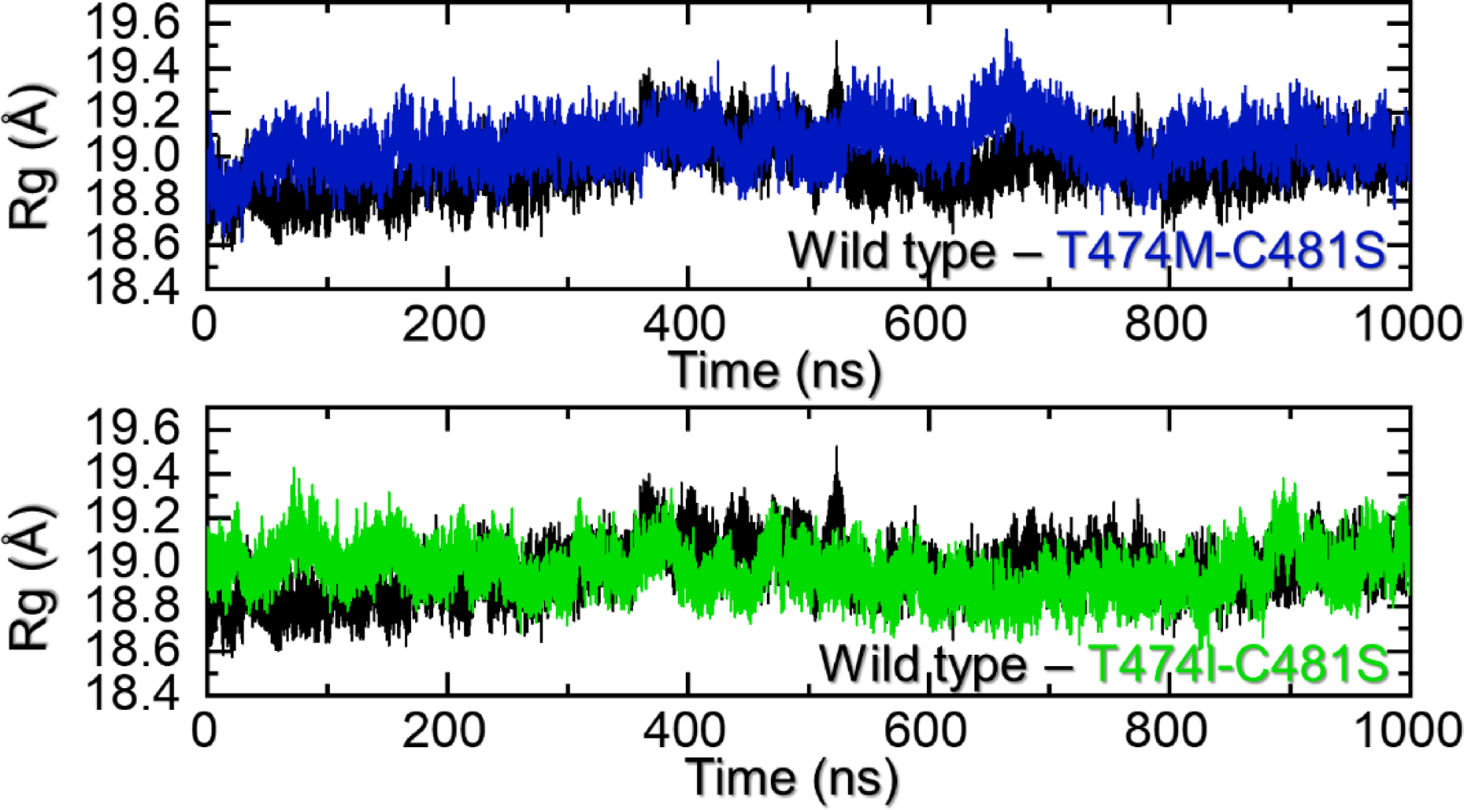
Structural compactness analysis of the WT and mutant complexes. **(a)** shows the Rg graphs for the WT and the T474M-C481S mutant. while **(b)** shows the Rg graphs for the WT and the T474I-C481S mutant.

Analysis of the protein structural size using the Rg as a function of time is an important parameter to determine the binding and unbinding events. It has been widely used to decipher essential biological mechanisms required for pathogenesis or molecular function. As shown in Fig. 5a, the WT complex reported a more uniform behavior with an average Rg of 18.90 Å throughout the simulation. No significant structural perturbation was observed apart from a minor deviation due to the free movement of region 545–559, which aligns with the RMSD results. The results show that the WT maintained a more constrained dynamic structure, and minimal unbinding events are experienced by the small molecule bound to the active cavity. On the other hand, the T474M-C481S mutant had an increase and decrease pattern in the protein size, mainly attributed to the movement of 432–439 and 545–559 regions. Moreover, the movement of ligand molecules in the cavity makes it more vulnerable to dynamic changes. An average Rg for the T474M-C481S mutant was calculated to be 19.05 Å, which shows the opening of the binding cavity with the movement of the aforementioned regions and thus causes an increase in the size of the protein. The Rg graph for the T474M-C481S mutant is found in Fig. 5a. In addition, the Rg pattern for the T474I-C481S mutant exhibited a wave-like behavior during the simulation. However, the Rg was maintained at a similar level for the WT. The average Rg for the T474I-C481S mutant was calculated to be 18.95 Å as represented in Fig. 5b. Overall. These observations indicate that the WT demonstrated a different behavior compared to the mutants, which consequently causes a reduction in the binding and ultimately leads to drug resistance.

### Residue flexibility analysis of the WT and mutant complexes

**Fig. 6:**
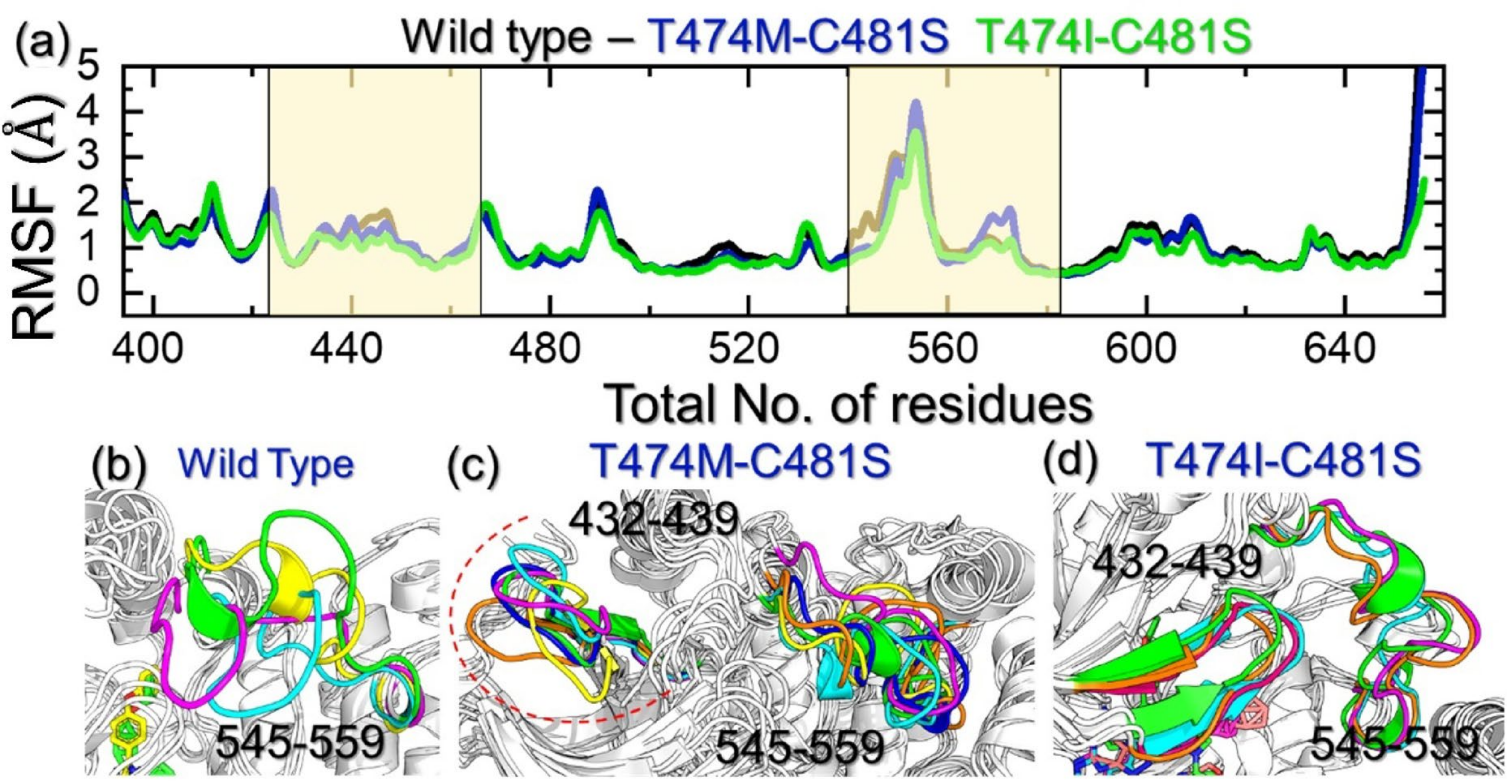
Structural flexibility analysis of the WT and mutant complexes.** (a)** shows the RMSF graphs for the WT, T474M-C481S, and T474I-C481S mutants, while **(b-d)** shows the highly dynamic regions in the WT and the mutants.

The determination of the residue flexibility is important in deciphering the role of key amino acids in ligand recognition, molecular catalysis, protein communication, and disease potential. It has been a widely applicable method in determining the impact of a particular substitution on the structure and function of a protein. We determined the residue flexibility as root mean square fluctuation, and the data are presented in Fig. 6a. All the complexes demonstrated similar behavior to the WT with minimal fluctuation, except the regions 432–439 and 545–559, where higher fluctuations were recorded. These dynamically more flexible regions are located within the active site, and their flexibility plays a crucial role in facilitating drug release by creating additional space for the drug to exit the binding cavity. The highly dynamic regions were mapped onto the structures of each complex, revealing secondary structure transitions, including shifts from loops to helices and from helices to loops, occurring at the site between residues 545 and 559. These transitions result in functional variations and serve as a foundation for drug resistance. These findings show that these mutations alter the internal flexibility and thus cause a variation in the ibrutinib binding. In our simulations, both regions we focused on, residues 432–439 and 545–559, are located in functionally relevant regions of the BTK kinase domain. The segment 432–439 is located just next to the β3-αC loop, which is responsible for positioning the αC-helix and subsequently stabilizing the conserved Glu-Kys salt bridge that is necessary for activation of the kinase. Residues 545–559 are part of the activation loop (A-loop), which has long been recognized as an important region for substrate specificity and catalytic activity. Therefore, instabilities in these loops are of functional relevance as they could modify the conformational landscape for active site geometry, ATP binding, and inhibitor binding. In the T474M-C481S and T474I-C481S mutants, our simulations showed that the loss of covalent anchoring C481 propagates into increased mobility and conformational changes/flexibility in these loops, thereby increasing room in the binding pocket, which limits the ability for ibrutinib to bind with meaningful strong affinity. Thus, the loop instabilities we observed are functionally linked to resistance, not solely in terms of structural interpretation but importantly in functional terms of inhibitor binding while still displaying basal kinase activity. The highly dynamic regions in each complex are shown in Fig. 6b and c, and 6d.

### Hydrogen bonding analysis

**Fig. 7:**
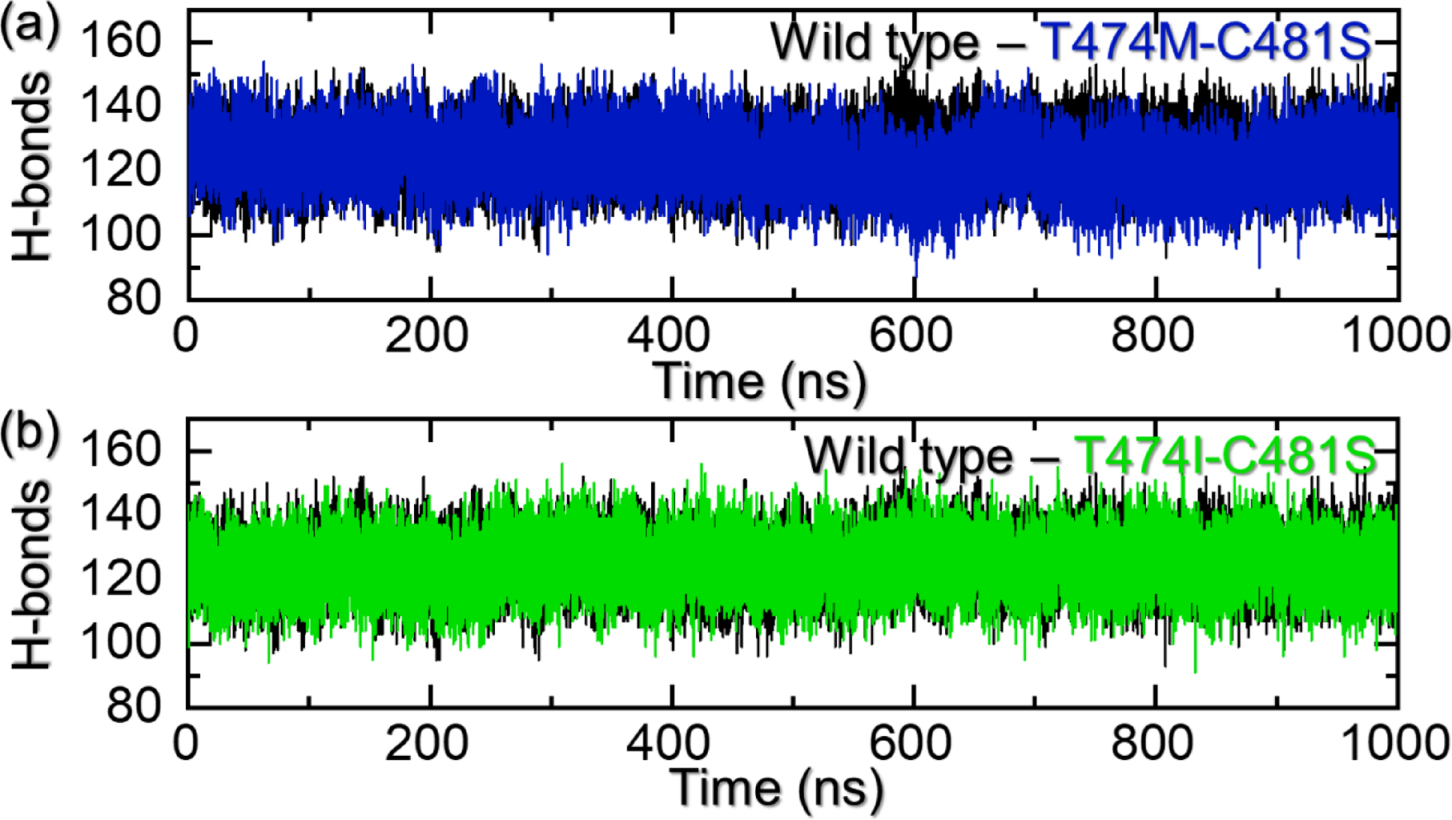
Hydrogen bonding analysis of the WT and mutant complexes. **(a)** shows the hydrogen bonding graphs for the WT, T474M-C481S, while (b) shows the hydrogen bonding graphs for the WT mutant.

The formation of macromolecular complexes is influenced by various factors, and crucial roles are played by hydrogen bonding and hydrophobic contacts in connecting proteins. Water molecules within the protein interface are particularly important, as they establish hydrogen bonds with residues, thereby enhancing the overall stability of the complex^43^. Despite advancements, the processes regulating molecular complexes and their impact on hydrogen bonding are not completely understood^44^. The role of hydrogen bonds in shaping molecular complexes has been a long-standing question, and the underlying mechanism is still not well understood^45,46^. Understanding the patterns of hydrogen bonding in molecular interactions is essential. Predicting hydrogen bonding provides valuable insights into the strength of association between two molecules. This knowledge is vital for understanding the mechanisms behind diseases, recognition, and resistance due to different mutations, as well as catalytic processes. Therefore, we calculated the total number of hydrogen bonds by analyzing the molecular simulation trajectory. The average number of hydrogen bonds in each complex determined the binding strength of ibrutinib. For instance, the WT reported 125 average hydrogen bonds, while the T474M-C481S and T474I-C481S reported 122 hydrogen bonds, respectively. This shows that due to these mutations, the number of hydrogen bonds has reduced significantly, which is due to the free movement of the ligand in the binding cavity and the inaccessibility of one tail or another. The hydrogen bonding graphs for the WT and mutants are given in Fig. 7a and b.

### Binding free energy calculation

Accurate estimation of the binding poses predicted by the docking simulation can be re-evaluated by using the binding free energy calculation. In contrast to the rational alchemical approach, these methods, i.e., MM-GBSA and MM-PBSA, are more accurate, computationally inexpensive, and up to date. They have been widely used to determine the binding efficiency of small molecules or variations caused by natural substitutions. Considering the implications of these approaches in accurately predicting drug resistance patterns, we used these approaches to calculate the binding free energy of the WT and mutant systems. Using the MM-GBSA approach, the WT reported a vdW of −66.58 ± 0.06 kcal/mol and an electrostatic energy of −6.07 ± 0.03 kcal/mol. On the other hand, the T474M-C481S and T474I-C48 mutants had vdW values of −62.96 ± 0.14 kcal/mol and − 58.38 ± 0.13 kcal/mol, respectively. This shows a significant decline in the vdW in the mutant complexes compared to the WT. Moreover, the electrostatic energy for T474M-C481S and T474I-C481S was calculated to be −0.21 ± 0.07 kcal/mol, and − 5.91 ± 0.05 kcal/mol, respectively. The T474M-C481S complex showed a significant decline in the electrostatic energy compared to the WT. From these data, it can be observed that each mutant system gains free energy in the gas phase but not in the solvent. These findings suggest that the ligand binding is thermodynamically favored in terms of enthalpy, primarily owing to advantageous interactions in the gas phase. However, it is disfavored in terms of entropy due to the unfavorable effects of solvation. To provide conclusive evidence, the total binding free energy (TBE) for the WT and mutant systems was calculated and summarized in Table 1. The WT had a TBE of −60.33 ± 0.06 kcal/mol, while T474M-C481S and T474M-C481S mutants reported TBE values of −53.18 ± 0.12 kcal/mol and − 49.12 ± 0.10 kcal/mol, respectively. It can be noted that a significant decline in the TBE values occurred in each complex due to these mutations, thereby reducing the pharmacological potential of ibrutinib. The MM-GBSA decomposition highlights van der Waals interactions (VDWAALS) as the primary stabilizing factor, with WT showing the strongest contribution (–66.58 kcal/mol). Electrostatics (EEL) are nearly abolished in T474M–C481S, suggesting this mutation disrupts charge complementarity, while solvation penalties (EGB + ESURF) partially offset binding in all complexes. Overall, the net binding free energy follows the order WT > T474M–C481S > T474I–C481S, consistent with progressive weakening of drug affinity in the mutants.

**Table 1:** Total binding free energy (TBE) using the MM-GBSA approach. All the results are calculated in kcal/mol. The total binding free energies are shown in bold in the table.

Parameters	MM-GBSA		
	WT	T474M-C481S	T474I-C481S
VDWAALS	−66.58 ± 0.06	−62.96 ± 0.14	−58.38 ± 0.13
EEL	−6.07 ± 0.03	−0.21 ± 0.07	−5.91 ± 0.05
EGB	19.78 ± 0.03	17.33 ± 0.07	22.17 ± 0.08
ESURF	−7.46 ± 0.00	−7.32 ± 0.01	−7.00 ± 0.01
DELTA G Gas	−72.66 ± 0.07	−63.18 ± 0.15	−64.29 ± 0.15
DELTA G Solv	12.32 ± 0.03	10.00 ± 0.07	15.16 ± 0.07
DELTA G	**−60.33 ± 0.06**	**−53.18 ± 0.12**	**−49.12 ± 0.10**

The TBE was also calculated using the MM-PBSA approach, and the results are summarized in Table 2. As shown in Table 2, similar values for vdW and electrostatic energies are reported compared to those determined with the MM-GBSA approach, indicating this represents a cross-validation of results. The results from the MM-PBSA approach also demonstrated that the binding is thermodynamically favorable in the gas phase but not in the solvent. Moreover, the TBE demonstrated a similar behavior as per what was observed in MM-GBSA results. The WT demonstrated a stronger binding affinity with a TBE of −42.65 ± 0.08 kcal/mol compared to the T474M-C481S mutant. Specifically, the T474M-C481S mutant exhibited a TBE of −38.81 ± 0.18 kcal/mol, indicating a slightly weaker binding affinity, while the T474I-C481S double mutant displayed the lowest affinity with a TBE of −33.04 ± 0.13 kcal/mol. These results underline the significant impact of mutations T474M and C481S on the binding free energy, highlighting the importance of these residues in the ibrutinib-BTK interactions. In the MM-PBSA results, van der Waals interactions again dominate binding, but are weakened in both mutants. Electrostatics are reduced in T474M–C481S, while polar solvation (EPB) strongly destabilizes T474I–C481S (+ 35.73 kcal/mol), consistent with its weakest overall binding. The trend in net ΔGbind (WT < T474M–C481S < T474I–C481S, i.e., most negative to least negative) clearly supports the experimental observation of reduced affinity for double mutants. Although solvation penalties and entropic costs oppose binding, the favorable gas-phase van der Waals and electrostatic interactions (enthalpic contributions) are sufficiently strong to outweigh these unfavorable terms.

**Table 2:** Total binding free energy (TBE) using the MM-PBSA approach. All the results are calculated in kcal/mol. The total binding free energies are shown in bold in the table.

Parameters	MM-PBSA		
	WT	T474M-C481S	T474I-C481S
VDWAALS	−66.58 ± 0.06	−62.96 ± 0.14	−58.38 ± 0.13
EEL	−6.07 ± 0.03	−0.21 ± 0.07	−5.91 ± 0.05
EPB	34.74 ± 0.08	29.00 ± 0.16	35.73 ± 0.20
ENPOLAR	−4.74 ± 0.00	−4.64 ± 0.00	−4.49 ± 0.00
DELTA G Gas	−70.80 ± 0.82	−63.18 ± 0.15	−64.29 ± 0.15
DELTA G Solv	29.68 ± 0.08	24.36 ± 0.16	31.24 ± 0.20
DELTA G	**−42.65 ± 0.08**	**−38.81 ± 0.18**	**−33.04 ± 0.13**

Beyond the diminished binding of ibrutinib to mutant BTK, it’s also worth noting that ibrutinib is not a selective inhibitor of BTK. Other kinases have been reported to inhibit, including ITK (interleukin-2–inducible T-cell kinase), TEC, BMX, EGFR, ERBB2, and JAK3. This broader inhibition of kinases both has therapeutic benefit, as well as causes side effects; for example, inhibiting ITK in T-cells skews the T-cell response toward a Th1 response that enhances anti-tumor immunity, further providing potential clinical benefit in hematological malignancies^47^, Inhibition of TEC family kinases, such as BMX, appears to result in vascular responses, which are a complication of ibrutinib therapy, and include EGFR and ERBB2 activity, are likely responsible for off-target toxicities, including rash and diarrhea^48,49^. Mechanistically, these additional targets show that while T474M and C481S amino acid substitutions in BTK diminish the binding interactions and decrease the effects of ibrutinib, the compound may still exert some limited biologic activity from its effects on other kinases. This phenomenon may help to explain the limited residual responses in some patients with resistance that contain BTK mutations. Importantly, this further highlights the critical imperative of next-generation BTK inhibitors to have enhanced inhibition, selectivity, and potency for resistant mutants (acalanbrutinib and zanubrutinib) and limited off-target interactions that might be associated with toxicity. All of these studies strengthen the structural basis of drug resistance in BTK and underscore the clinical consequences of off-target inhibition of kinases related to both the therapeutic efficacy and adverse events in those patients with ibrutinib.

### Principal component analysis (PCA)

**Fig. 8:**
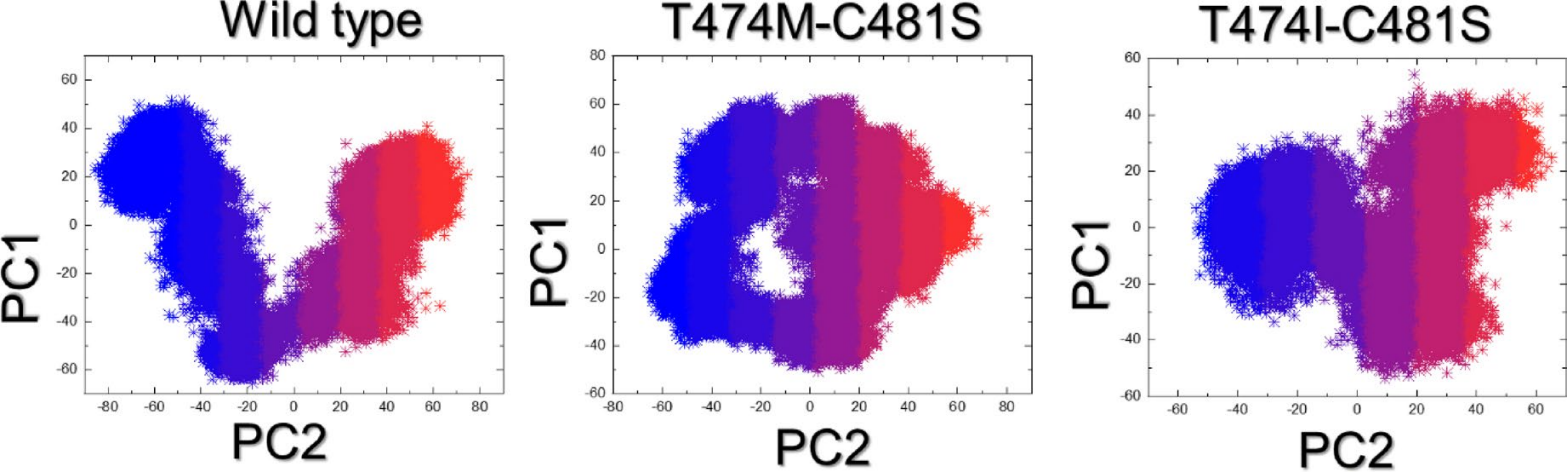
PCA analysis of the WT and mutant complexes. The two conformational states are represented with the blue and orange colors.

To assess the motion trajectories and categorize them based on the achieved conformational states, PCA analysis was conducted on each trajectory, as illustrated in Fig. 8. The findings revealed that the majority of the WT exhibited non-constrained motion, with notable distribution along the axis. In contrast, mutants displayed constrained motion, indicating a decrease in motion and a restriction to narrower conformational ensembles. Such restriction reflects reduced conformational adaptability, which is essential for maintaining stable inhibitor interactions. This implies that the double mutants may compromise the functional plasticity of BTK, thereby perturbing the ability of ibrutinib to remain tightly engaged within the binding pocket.

### Free energy landscape analysis (FEL)

**Fig. 9:**
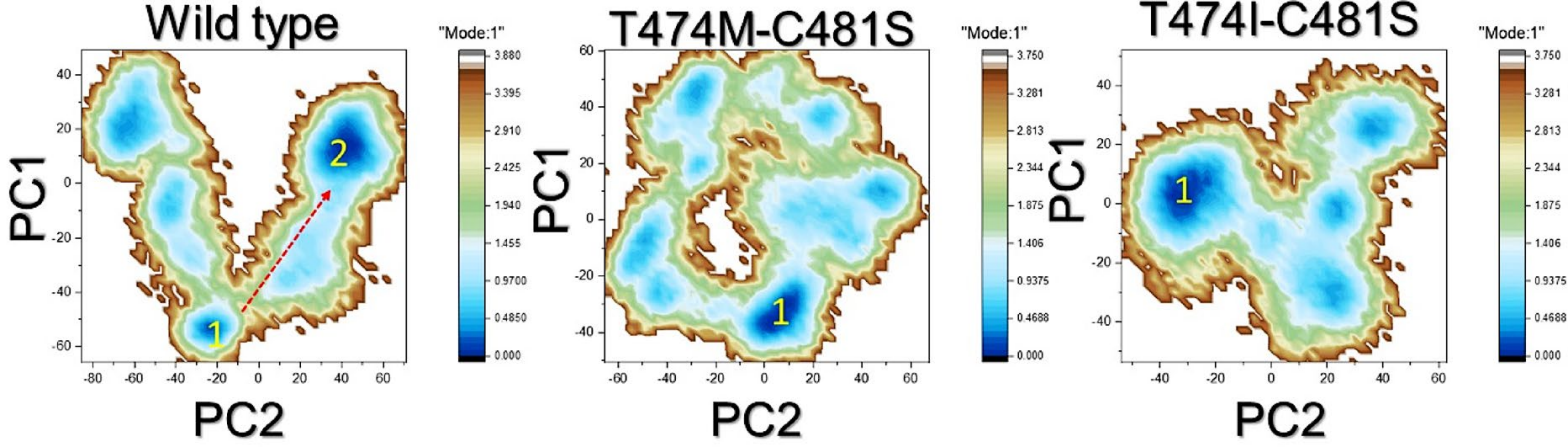
Prussian blue iron stains of GBM39 tumors after theranostic therapy. (A,C,E) Representative Prussian blue (PB)stained tumor sections from the combination therapy group, monotherapy group, and control group at 48 h after intravenous injections. Scale bar: 100 μm. (G) Quantitative analysis of Prussian blue-positive staining area. Box plots illustrate the proportion of positively stained areas relative to the total tumor area (%) for each group (n=4 mice in each group). Statistical comparison of PB-positive staining between the combination therapy group and monotherapy group was performed using the Mann–Whitney U test. The control group showed no detectable PB staining and was excluded from statistical analysis. (B,D,F) Representative hematoxylin and eosin (H&E) stained tumor sections from the combination therapy, monotherapy, and control groups, respectively. Scale bar: 100 μm. We did not observe any significant intra-tumoral hemorrhage in response to TNP treatment.

The free energy landscape is a crucial concept that provides valuable insights into the behavior and stability of a protein or any other biomolecular system. It is a representation of the energy landscape of the system in a multi-dimensional space defined by various degrees of freedom, such as atomic coordinates and bond angles. Understanding the free energy landscape is essential because it helps to uncover the stable states and transition pathways of the biomolecular system. The WT presented multiple energy conformational states, whereas the mutants displayed only a single, lower-energy conformational state. The presence of multiple minima in the WT reflects structural heterogeneity and the ability to adopt conformations conducive to drug binding. In contrast, the mutant landscapes collapse into a single basin, indicating loss of conformational diversity and entrapment in suboptimal states that weaken inhibitor accommodation. Collectively, these results suggest that mutation-driven narrowing of the conformational landscape directly contributes to drug resistance. This observation suggests that these mutations have induced distinct effects, consequently leading to variations in the binding of ibrutinib. The FEL results are shown in Fig. 9.

## Conclusions

This study employed molecular simulation approaches to investigate the effects of T474M-C481S and T474I-C481S mutants on the structure of BTK and their influence on the binding of ibrutinib. The analysis revealed that essential interactions, such as hydrogen bonds, predominantly disrupt structural integrity, specifically in the regions spanning from 432 to 439 and 545 to 559. In addition, the drug’s ability to move inside the binding cavity restricts the accessibility to the active residue, resulting in a decrease in binding affinity. These findings were further substantiated by a marked decrease in the total binding free energy, emphasizing the significance of these interactions. Furthermore, our study utilized PCA and FEL techniques to reveal discrepancies in the internal dynamics and energy landscape of each mutant complex. While our investigation yields important mechanistic information generated via extensive molecular dynamics simulations and binding free energy calculations, inherent limitations are understood in the context of only computational methodologies assessed. Force-field approximations limited conformational sampling, and dependence on modeled structures all add uncertainty to the study, and the in-silico binding affinities cannot be relied upon as replacements for experimentally determined binding measurements. Notably, the BTK double mutants (T474M–C481S and T474I–C481S) have already been demonstrated experimentally to strongly confer resistance to covalent BTK inhibitors, while the specific structural mechanisms of resistance remain incompletely defined. As such, this study offers insight into predicting how these mutations alter drug–protein binding at atomic resolution, thereby providing hypotheses that can inform and prioritize future biochemical and structural validation experiments. Therefore, further binding assays and crystallography techniques will provide more evidence regarding the precise mechanism and future drug development. Taken together, these results highlight that the loss of covalent anchoring at C481, coupled with loop flexibility, is the key driver of resistance. Beyond defining this mechanism, our findings underscore the broader relevance of integrating dynamics-based insights into next-generation BTK inhibitor design. By considering both structural adaptability and alternative binding strategies, such computational insights may contribute to precision-medicine approaches and guide the development of therapies that remain effective even against clinically resistant BTK variants.

## Supplementary Information

Below is the link to the electronic supplementary material.

Supplementary Material 1

## Data Availability

The data will be made available on reasonable demand. Some of the data can be obtained from RCSB using the accession ID given in the manuscript.
