# Correction: Microsecond simulations to investigate the structural mechanism of super-resistant double mutations in BTK to the covalent inhibitor ibrutinib in multiple leukemia

**DOI:** 10.1038/s41598-026-36843-1

**Published:** 2026-01-20

**Authors:** Abbas Khan, Syed Shujait Ali, Muhammad Ammar Zahid, Fahad M. Alshabrmi, Raed M. Al-Zoubi, Mohanad Shkoor, Anwar Mohammmad, Dong-Qing Wei, Abdelali Agouni

**Affiliations:** 1 Department of Pharmaceutical Sciences, College of Pharmacy, QU Health, Qatar University, P.O. Box 2713, Doha, Qatar; 2 Center for Biotechnology and Microbiology, University of Swat, Swat, Khyber Pakhtunkhwa Pakistan; 3 Department of Medical Laboratories, College of Applied Medical Sciences, Qassim University, 51452 Buraydah, Saudi Arabia; 4 Surgical Research Section, Department of Surgery, Hamad Medical Corporation, Doha, Qatar; 5 Department of Biomedical Sciences, College of Health Sciences, QU Health, Qatar University, P.O. Box 2713, Doha, Qatar; 6 Department of Chemistry, Jordan University of Science and Technology, P.O. Box 3030, Irbid, 22110 Jordan; 7 Department of Chemistry and Earth Sciences, College of Arts and Science, Qatar University, P.O. Box 2713, Doha, Qatar; 8 Precision Health Analysis Unit, Translational Research, Dasman Diabetes institute, Dasman, Kuwait; 9 Department of Biostatistics and Bioinformatics, College of Life Sciences and Biotechnology, Shanghai Jiao Tong University, Shanghai, P.R. China

Correction to: *Scientific Reports* 10.1038/s41598-025-24745-7, published online 20 November 2025

In the original version of this Article Abdelali Agouni was incorrectly affiliated with Affiliation 9: “Department of Biostatistics and Bioinformatics, College of Life Sciences and Biotechnology, Shanghai Jiao Tong University, Shanghai, P.R. China.” The author is affiliated only with “Affiliation 1: Department of Pharmaceutical Sciences, College of Pharmacy, QU Health, Qatar University, P.O. Box 2713, Doha, Qatar”. The original Article has been corrected.

