# Supplementary material for: Microsecond simulations to investigate the structural mechanism of super-resistant double mutations in BTK to the covalent inhibitor ibrutinib in multiple leukemia
Source: Sci Rep. 2025 Nov 20;15:40934. doi: 10.1038/s41598-025-24745-7 (PMC12635121; doi:10.1038/s41598-025-24745-7)
Supplement: Supplementary file 1 — Supplementary Material 1 [file 41598_2025_24745_MOESM1_ESM.docx]

**Supplementary Information**

**Microsecond simulations to investigate the structural mechanism of super-resistant double mutations in BTK to the covalent inhibitor Ibrutinib in multiple leukemia**

Abbas Khan^1^, Syed Shujait Ali^2^, Muhammad Ammar Zahid^1^, Fahad M Alshabrmi^3^, Raed M. Al-Zoubi^4,5,6^, Mohanad Shkoor^7^, Anwar Mohammmad^8^, Dong-Qing Wei^9^, Abdelali Agouni^1,9*^

1. Department of Pharmaceutical Sciences, College of Pharmacy, QU Health, Qatar University, P.O. Box 2713, Doha, Qatar.
2. Center for Biotechnology and Microbiology, University of Swat, Swat, Khyber Pakhtunkhwa, Pakistan.
3. Department of Medical Laboratories, College of Applied Medical Sciences, Qassim University, Buraydah 51452, Saudi Arabia.
4. Surgical Research Section, Department of Surgery, Hamad Medical Corporation, Doha, Qatar
5. Department of Biomedical Sciences, College of Health Sciences, QU Health, Qatar University, P.O. Box 2713, Doha, Qatar.
6. Department of Chemistry, Jordan University of Science and Technology, P.O. Box 3030, Irbid, 22110, Jordan
7. Department of Chemistry, College of Arts and Science, Qatar University, P.O. Box 2713, Doha, Qatar.
8. Department of Biochemistry and Molecular Biology, Dasman Diabetes institute, Dasman, Kuwait.
9. Department of Biostatistics and Bioinformatics, College of Life Sciences and Biotechnology, Shanghai Jiao Tong University, Shanghai, P.R China.

**Materials and Methods**

**Post-Simulation analysis and Binding Free Energy Calculation**

For calculating RMSD the following mathematical expression was used;

$RMSD= \sqrt{\frac{1}{N} \sum_{i=1}^{N} \delta_{i}^{2}}$ (i)

In the above equation, δi = represents the distance of atom *i* from the reference or average position of N equivalent atoms. Usually, this is employed for the heavy atoms in the backbone, such as C, N, O, and Cα, or occasionally only for the Cα atoms.


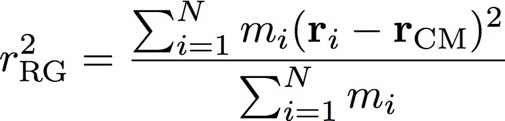
 (ii)

Where:

- **r**_i_ determines the position of the atom at index *i,*
- *m*_i_ shows the mass of the atom at index *i,*
- **r**_CM_ represents the center of mass,
- N determines the number of atoms being counted, and
- r^2^_RG_ is the square of the radius of gyration.

For the residues’ flexibility analysis, the following mathematical expression was used.

$B= \frac{{8\pi}^{2}}{3}\langle\Delta r^{2}\rangle$ (iii)

Where:

B stands for B-factor, and

⟨Δr^2^⟩ represents the mean square deviation (i.e., ⟨Δr^2^⟩ = RMSD^2^).

By rearranging the above equation and accounting for 3 spatial dimensions, we can obtain the RMSF like so:

$RMSF= \sqrt{\frac{3B}{{8\pi}^{2}}}$ (iv)

This equation can be used to determine the contribution of interaction in the complex and can be expressed as;

$G= E_{Molecular Mechanics}- G_{solvated}- TS$ (vi)

This equation can be further restructured to calculate the specific energy term.

${\Delta G}_{bind}= {\Delta E}_{Molecular Mechanics}+ {\Delta G}_{solvated}- \Delta TS= {\Delta G}_{vaccum} + {\Delta G}_{solvated}$(vii)

${\Delta E}_{Molecular Mechanics}= {\Delta E}_{int}+ {\Delta E}_{electrostatic} + {\Delta E}_{vdW}$ (viii)

$\Delta G_{solvated}= \Delta G_{Generalized born}+\Delta G_{surface area}$ (ix)

$\Delta G_{surface area}= \gamma.SASA+b$ (x)

$\Delta G_{vaccum}= {\Delta E}_{Molecular Mechanics}-T\Delta S$ (xi)

The total binding energy is determined by the contribution of each of the terms mentioned above. Specifically, the free energy of ligand-protein/protein-protein or protein/nucleic acid total binding is represented by *ΔG_bind_*. The total gas phase energy, which is the sum of *ΔE_internal_*, *ΔE_electrostatic_*, and *ΔE_vdw_,* is reflected in *ΔE_MM_*. The sum of polar (*ΔG_PB/GB_*) and non-polar (*ΔG_SA_*) contributions to solvation is represented by *ΔG_sol_*. The conformational binding entropy, typically calculated through normal-mode analysis, is represented by *-TΔS*. The internal energy arising from various bonds, angles, and dihedral in the molecular mechanics (MM) force field is reflected in *ΔE_internal_*. *ΔE_electrostatic_* and *ΔE_vdw_* are the electrostatic and van der Waals energies calculated using MM, while ΔG_PB/GB_ represents the polar contribution to the solvation-free energy, calculated using Poisson–Boltzmann (PB) or generalized Born (GB) methods. *ΔG_SA_* is the nonpolar solvation-free energy, usually calculated using a linear function of the solvent-accessible surface area (SASA). The conformational entropy and enthalpy change were also calculated to revalidate the binding accuracy and reveal more information.

**Essential Dynamics**

When given a collection of n-dimensional vectors (xi), the covariance matrix (C) may be calculated using the following mathematical representation:

$C= \frac{1}{N} x \sum\left( i=1 to N \right)\left[ {(x}_{i}- \mu\right) x (x_{i}- \mu)^{T}]$ (xii)

Where:

N = number of vectors,

μ = mean vector, while

T designates the transpose operation.

The following equation can be used to obtain the eigenvectors (V) and eigenvalues (λ) of the covariance matrix C.

$C x V = \lambda x V$ (xiii)

Where V stands for the eigenvector matrix and λ for the diagonal matrix of eigenvalues. The principal components of the system are represented by the eigenvectors with the highest matching eigenvalues. We also computed the two PCs, PC1 and PC2, employing the unsupervised PCA method to comprehend the fundamental movements in our systems. The CPPTRAJ module in AMBER21 was utilized for this task, taking into account the whole simulation trajectory, or 10,000 frames for each system. The spatial covariance matrix was calculated using the eigenvectors and their atomic coordinates. A diagonal eigenvalue matrix was created using an orthogonal coordinate transformation. The system's primary components were identified using the eigenvectors and eigenvalues, and they were then used to illustrate the important movements seen.
